# Improving Patient-Centered Care for Young People in General Practice With a Codesigned Screening App: Mixed Methods Study

**DOI:** 10.2196/mhealth.7816

**Published:** 2017-08-11

**Authors:** Marianne Julie Webb, Greg Wadley, Lena Amanda Sanci

**Affiliations:** ^1^ Department of General Practice, Melbourne Medical School Faculty of Medicine University of Melbourne Parkville Australia; ^2^ School of Computing and Information Systems University of Melbourne Parkville Australia

**Keywords:** adolescent, needs assessment, general practice, primary prevention, health behavior, health information technology, patient-centered care

## Abstract

**Background:**

Despite experiencing a high prevalence and co-occurrence of mental health disorders and health-compromising behaviors, young people tend not to seek professional help for these concerns. However, they do regularly attend primary care, making primary care providers ideally situated to identify and discuss mental health and lifestyle issues as part of young people’s routine health care.

**Objective:**

The aim was to investigate whether using a codesigned health and lifestyle-screening app, Check Up GP, in general practice influenced young people’s assessment of the quality of their care (measures of patient-centered care and youth friendliness), and their disclosure of sensitive issues. In addition, this study aimed to explore young people’s acceptance and experience of using a screening app during regular health care.

**Methods:**

This was a mixed methods implementation study of Check Up GP with young people aged 14 to 25 years attending a general practice clinic in urban Melbourne, Australia. A 1-month treatment-as-usual group was compared to a 2-month intervention group in which young people and their general practitioners (GPs) used Check Up GP. Young people in both groups completed an exit survey immediately after their consultation about disclosure, patient-centered and youth-friendly care, and judgment. In addition, participants in the intervention group were surveyed about app acceptability and usability and their willingness to use it again. Semistructured interviews with participants in the intervention group expanded on themes covered in the survey.

**Results:**

The exit survey was completed by 30 young people in the treatment-as-usual group and 85 young people in the intervention group. Young people using Check Up GP reported greater disclosure of health issues (*P*<.001), and rated their GP higher in patient-centered care: communication and partnership (*P*=.01), personal relationship (*P*=.01), health promotion (*P*=.03), and interest in effect on life (*P*<.001). No differences were found on core indicators of youth-friendly care: trust, level of comfort, expectations met, and time to ask questions. In all, 86% (73/85) of young people felt the app was a “good idea” and only 1% (1/85) thought it a “bad idea.” Thematic analysis of qualitative interviews with 14 participants found that Check Up GP created scope to address unmet health needs and increased sense of preparedness, with use moderated by honesty, motivation, app content and functionality, and app administration.

**Conclusions:**

Integrating a health and lifestyle-screening app into face-to-face care can enrich young people’s experience of seeing their GP, create scope to identify and address unmet health needs, and increase patient-centered care. Further research is needed to investigate the effect of using a health and lifestyle-screening app in a diverse range of clinic types and settings, and with a diverse range of GPs and youth.

## Introduction

Adolescence and young adulthood are periods of major transition in physical, cognitive, social, and emotional development along the journey from childhood to adulthood [1]. These are also periods when mental health disorders and health-compromising behaviors emerge [1]. Worldwide, substance use, poor diet, lack of exercise, and mental health disorders are the leading risk factors for years lost due to ill health, disability, or early death for young people [2]. These disorders and behaviors tend to co-occur [3,4] and persist into adulthood [5-7].

Despite experiencing a high prevalence and co-occurrence of mental health disorders and lifestyle issues, young people do not usually seek professional help for these concerns [8]. Yet, they do regularly attend primary care, usually to address physiological concerns [9,10], making primary care providers (PCPs) ideally situated to opportunistically discuss mental health and lifestyle issues, and to provide health promotion and early intervention as needed. Both young people and PCPs report wanting to have these discussions [11,12], yet seldom do so [10,12,13], even during well-child visits in the United States, which are appointments dedicated to screening for health and lifestyle issues and providing preventive health care [14].

Patient-centered care is a theoretical concept proposing that for therapeutic benefits patients need to be actively engaged in their care, with a focus on communication, partnership, and health promotion in the doctor-patient consultation [15]. Patient-centered care has been associated with improved symptom burden, satisfaction, and enablement [16]. Although there is limited research in youth populations, evidence suggests that young people prefer a patient-centered approach, and taking an active partnership role in decision making over a more passive role [17]. Despite this, many young people do not receive patient-centered care, even though it is associated with higher ratings of quality care [18].

Similar to patient-centered care, youth-friendly care is an evidence-based theoretical framework for delivering quality health care. However, youth-friendly care looks specifically at the indicators of quality service delivery for adolescents and young adults. Ambresin et al [19] have developed a theoretical framework for youth-friendly care at both the clinic and individual level: accessibility of health care, staff attitude (trustworthy, supportive, respectful), communication, medical competency, evidence-based guideline-driven care (confidentiality, comprehensive care), age-appropriate environment (privacy, teen-orientated health information), involvement in health care, and health outcomes. Thus, the patient-centered and youth-friendly care theoretical frameworks can provide the foundations for designing and evaluating technological interventions designed to improve young people’s engagement with health care.

Regular screening of young people for a range of health and lifestyle issues is recommended by peak professional bodies [20,21] and, along with subsequent intervention, may improve health outcomes [22]. It is known that screening via technology increases disclosure when compared with paper [23,24] and face-to-face [25,26] formats. Furthermore, young people prefer initially disclosing health information via technology rather than face-to-face, even when they know the results will be reviewed by their practitioner [27,28]. Previous studies have validated technology-based health and lifestyle-screening tools for young people [29,30].

Evidence in non-youth populations suggests that technology-based screening improves patient-centered care [31,32]; however, evidence is limited for the impact on young people’s patient-centered and youth-friendly care. One study by Gadomski et al [33] undertook an analysis of audiotaped consultations and found that technology-based screening tool use increased doctor engagement and discussion of psychosocial and mental issues, without affecting partnership or rapport. However, this study did not interview young people about their experience of care. Another study by Olson et al [34] found that young people using a technology-based screening tool were more likely to feel that they were listened to carefully, that the discussion was confidential, and to feel more satisfied. Other core elements of youth-friendly care, such as trust and respect, and key elements of patient-centered care, such as health prevention and promotion and shared power, remain unexplored. Both the studies of Gadomski et al and Olson et al were limited to young people aged 19 years or younger and attending well-child visits [33,34]. More evidence is needed about the use of technology-based screening during other occasions of care because less than 50% of young people attend well-child consultations [35,36]; therefore, most are not receiving preventive care. Further, young adults aged 18 to 25 years have a higher prevalence of significant health risks, such as sexually transmitted infections, substance use, and mental health problems [37], and have more unmet health needs [38] compared to younger adolescents. Hence, testing of the utility of technology-based screening is also required in this group.

Guidelines recommend annual preventive screening [20,21]; however, there is a paucity of research on whether young people are willing to use technology-based screening tools on a regular basis and, if so, how often. Although one study in a hospital-based adolescent and young adult clinic found that 84% of young people would be willing to complete a screening tool once a year, participants responded before their consultation [39]. It is possible their responses might be different after the experience of using the tool with their practitioner and at a practice not servicing only youth.

The aim of this study was to investigate how using a codesigned health and lifestyle-screening app, Check Up GP *,* based on the theoretical principles of patient-centered and youth-friendly care, in an Australian general practice influenced young people’s assessment of care and their engagement through disclosure of sensitive issues. We were also interested in understanding young people’s acceptance and experience of using a screening app as part of their regular health care.

This study addresses several aspects of the evidence gap by first conducting an in-depth mixed methods analysis of young people’s experience of using a technology-based screening tool incorporated opportunistically into routine health care visits rather than well visits, and second, by including young people aged 14 to 25 years in the study. Furthermore, Australian general practice, although being the most commonly accessed form of primary health care by Australian youth, caters for the population across the life span rather than being a youth-specific service, hence this study also provides evidence relevant to generalist health care settings.

## Methods

### Study Design and Setting

We conducted a mixed methods implementation study in 2016, comparing a 1-month treatment-as-usual (TAU) phase with a 2-month intervention phase. In the intervention phase, Check Up GP was integrated into the routine care of young people attending a general practice. The length of the study was decided by mutual agreement between the clinic and researchers prior to the commencement. Ethics approval was obtained from the University of Melbourne (Ethics ID #1544281).

One general practice clinic was recruited through the Victorian Primary Care Practice-Based Research Network, managed by the Department of General Practice at the University of Melbourne. The clinic is a large general practitioner (GP)-owned and operated practice located in an area of relative socioeconomic advantage in urban Melbourne, Australia, staffed by 12 GPs, a practice manager, a reception coordinator, and eight receptionists. Open 365 days a year, the practice is funded by a patient copayment on top of the national health care basic amount.

### Participants

The four GP principal owners of the practice participated in the study, along with their patients aged 14 to 25 years attending for routine primary care during the study period. Patients were excluded from the study if their GP assessed that their patient was very unwell (eg, vomiting, weak, psychotic), unable to read/speak English, or if they were younger than 18 years of age and not a mature minor [40]. The practice support staff, being the practice manager, reception coordinator, and all eight receptionists, also participated. The practice support staff were responsible for administering Check Up GP to young people.

### Check Up GP App and Codesign Process

Based on the design needs identified in codesign workshops held with young people, GPs, practice support staff, and parents (reported previously [41]), we contracted a commercial software developer to build the health and lifestyle-screening tool, Check Up GP *.* To continue the codesign process, we recruited two reference groups representing the main end users of the tool; one of young people aged 14 to 25 years and another of GPs. These reference groups provided feedback and guidance on the design, content, and implementation of Check Up GP to ensure the final product reflected the requirements identified in the original codesign workshops. This process was also directly guided by the two theoretical frameworks, with each component of patient-centered care and youth-friendly care (described previously) being incorporated into Check Up GP. The resulting key technology and design features of Check Up GP are mapped onto each component of patient-centered care and youth-friendly care theoretical frameworks in Table 1.

Check Up GP consisted of two components: the questionnaire that patients answered and the summary report for GPs. The questionnaire was adapted from the HEEADSSS (Home environment, Education and employment, Eating, peer-related Activities, Drugs, Sexuality, Suicide and depression, Safety from injury and violence) preventive health framework for interviewing adolescents [42,43] recommended by the Royal Australian College of General Practitioners [21]. The framework covers the range of health, social, psychological, and physiological issues and behaviors contributing to the major burdens of disease for young people and suggests that they be raised and explored with young patients. We included validated screening tools in many of the HEEADSSS domains: eating disorders (SCOFF Questionnaire) [44], anxiety (Generalized Anxiety Disorder-2 [GAD-2]) [45], depression (Patient Health Questionnaire-2 [PHQ-2]) [46], and drugs and alcohol (CRAFFT screening test) [47]. At the beginning of the app, we included information about the research study and the purpose of the app, supplemented by a short video. Youth responses to the questions in the app in the format of a clinician summary report were immediately available to the GP via a secure website. The summary highlighted areas of concern, and strengths, along with tips on youth-friendly consultations and suggested actions to take on areas of concern including referral options, information, and resources. Screenshots of the youth and GP interfaces are included in Multimedia Appendix 1.

### Study Procedure and Measures

The study had three main phases: a TAU phase that consisted of a GP profile survey conducted at the start of data collection on GPs self-perceived ratings of consulting and communicating with youth, and youth “exit surveys” conducted at the practice of young people postconsultation with their GP; an intervention phase, that involved exit surveys of young people postconsultation after Check Up GP had been implemented; and a postintervention phase employing semistructured interviews with young people. The measures and procedure for each phase are described subsequently and summarized in Table 2.

**Table 1:** Key technology and design features of Check Up GP according to codesign workshop requirements and theoretical framework components.

Young people’s identified requirements	Theoretical framework (component)^a^	GPs’ identified requirements^a^	Theoretical framework (component)^a^
Link to Check Up GP sent via SMS at time of making the appointment	YFC (age-appropriate, accessibility)	Secure server	YFC (guideline-driven: confidentiality)
Secure log in	YFC (guideline-driven: confidentiality, accessibility)	Areas of concern/strengths displayed via traffic-light system from low risk (green) through to moderate (yellow) and high risk (red)	YFC (guideline-driven care), PCC (partnership, communication)
Choice of whether to complete on own device prior to attending appointment or on tablet in clinic waiting room immediately prior to appointment	YFC (age-appropriate; accessibility)	High level summary, with ability to expand for more detail	PCC (partnership, communication)
Youth-friendly language, design	YFC (age-appropriate, communication), PCC (communication)	Tips on youth-friendly practice including communication skills, negotiating a management plan	YFC (guideline-driven care, involvement in health care, communication, staff attitude), PCC (partnership, communication)
Ability to skip questions and flag issues for discussion	YFC (involvement in health care), PCC (partnership)	Suggested evidence-based/recommended for actions and health promotion, referrals to appropriate services and self-help apps	YFC (medical competency, guideline-driven, health outcomes), PCC (health promotion, partnership, communication)
		Ability to save as PDF for export to electronic health record	YFC (medical competency, guideline-driven)

^a^PCC: patient-centered care theoretical framework; PDF: portable document format; YFC: youth-friendly care theoretical framework.

#### Phase 1: Treatment As Usual

The GPs completed a brief paper-based profile survey of demographic information and self-rated their enthusiasm, knowledge, and confidence in consulting and communicating with youth. We then collected exit interviews from young people attending the clinic. During this phase, researchers approached young patients in the waiting room when they arrived and, if they were seeing a participating GP and aged 14 to 25 years, the researcher provided them with information about the study and invited them to participate. If they agreed to participate, the young patient was asked to take a form into their consultation for their GP to assess their eligibility to participate in the study. The patient handed the form back to the researcher on returning to the waiting room. After their consultation, each participant completed the short exit survey on a tablet in the waiting room, with consent provided at the start of the survey. As described in Table 2, this survey asked young people to self-rate levels of disclosure and to rate patient-centered and youth-friendly care and perceived judgment from their GP.

#### Phase 2: Intervention

Following the TAU phase, clinic staff were introduced to the Check Up GP app. The GPs and practice support staff were trained in administrating and using Check Up GP. This included showing a short video, which demonstrated how to administer and integrate the tool into the consultation with young people. We then met individually with each GP to ensure that they could easily access the online clinician summary on their computer and felt comfortable using it. At this time, two training points were stressed: (1) the importance of acknowledging all issues raised and, if time was not available to address them, to then organize a follow-up consultation and (2) to ask for time alone when discussing personal or sensitive issues if a parent was attending with the young person. These practice points were also available to GPs in the clinician interface of the app. We worked closely with GPs and practice support staff throughout the intervention phase to refine the app and its administration in rapid cycles of continuous quality improvement [51].

**Table 2:** Measures used in the treatment-as-usual (TAU), intervention, and postintervention phase.

Name of measure			Type, source, and content of measure		Method and phase of administration
**GP measures**					
	GP profile survey	Demographic information survey; age, gender, previous training in youth health		Paper questionnaire, TAU	
	Knowledge and confidence	Self-rated Likert scales (from 1=not at all to 7=extremely) from Sanci et al [48]; how knowledgeable and confident in consulting with young people aged 14-17, consulting with young people aged 18-25, consulting with male young people, consulting with female young people, communicating with young people, and exploring issues beyond the presenting problem		Paper questionnaire, TAU	
	Enthusiasm	Self-rated Likert scale (from 1=very unenthusiastic to 11=very enthusiastic) from Sanci et al [48]; enthusiasm for seeing young people		Paper questionnaire, TAU	
**Young people’s measures (completed post consultation)**					
	Disclosure	Self-rated Likert scales (from 1=no disclosure to 5=full disclosure, or not applicable) adapted^a^ from Bradford and Rickwood [25]; disclosure about home life, school/work, bullying/harassment, alcohol and drug use, sexual health and sexuality, safety, mood, addiction, stressful events, diet, exercise, sleep patterns, and risky behavior		Tablet questionnaire, TAU and intervention	
	Patient-centered care	Self-rated Likert scales (from 1=very strongly agree to 7=very strongly disagree), validated tool by Little et al [16]; a 21-item validated tool consisting of 5 components of patient-centered care: communication and partnership, personal relationship, health promotion, positive approach to diagnosis and prognosis, and interest in the effect on life		Tablet questionnaire, TAU and intervention	
	Youth-friendly care	Self-rated Likert scales, selected items from tool^b^ by Haller et al [49]; trust in the GP (from 1=poor to 5=excellent), whether expectations were met (from 1=strongly disagree to 4=strongly agree), whether there was enough time to ask questions (from 1=strongly disagree to 4=strongly agree), and level of comfort with GP (from 1=not at all to 5=very comfortable)		Tablet questionnaire, TAU and intervention	
	Fear of judgment	Self-rated Likert scales (from 1=not at all concerned to 5=very concerned) adapted^c^ from Bradford and Rickwood [25]; how worried young people were that the GP would think they were a bad person if they talked about everything they had been thinking, feeling, and doing; the GP would think there was something really wrong with them; they would learn things about themselves they didn’t want to know; they would lose control of their emotions; and that the GP would judge them		Tablet questionnaire, TAU and intervention	
	App acceptability	Self-rated Likert scales (from 1=not at all to 5=very much) from Bradford and Rickwood [25]; confidence of Check Up GP providing an accurate picture, comfort in disclosing personal information through Check Up GP, whether questions were difficult to understand, whether questions caused upset, and whether GP addressed issues raised		Tablet questionnaire, intervention	
	App usability	Self-rated Likert scales selected items from validated tool by Stoyanov et al [50]; how easy Check Up GP was to learn (from 1=very hard to 5=able to use immediately) and how good Check Up GP looked (from 1=no visual appeal to 5=very attractive)		Tablet questionnaire, intervention	
	Overall opinion and willingness to use again	Self-rated categorical responses; opinion of using Check Up GP (good idea, bad idea, don’t know), whether willing to use again (yes, no, and, if so, how often)		Tablet questionnaire, intervention	
	Youth interview	Semistructured interview; experience of using Check Up GP, how it was administrated and integrated into routine care, if they would like to use again in the future		Phone interview, audiotaped, postintervention	

^a^Adaption was replacing “therapist” with “doctor” and “I lied or misrepresented myself” with a “not applicable” option.

^b^English version of tool validated in Bosnian language.

^c^Adaption was replacing “therapist” with “doctor.”

Receptionists informed young people or parents about Check Up GP when they called the clinic to make an appointment. Receptionists then flagged the appointment in the clinical software so the reception coordinator would send the patient a short message service (SMS) text message containing the tool link. Patients could choose to complete Check Up GP before they arrived at the clinic or in the waiting room just prior to their appointment. When young patients arrived at the clinic, receptionists asked those aged 14 to 25 years who were patients of participating GPs if they had received the SMS text message and completed the app. Patients who had not completed the app were handed a tablet to complete it in the waiting room before their consultation. The receptionist made a note in the clinical software reminding the GP to review the young person’s responses prior to calling them into the consultation.

During the intervention phase, researchers approached young people in the waiting room to participate in the study and complete an exit survey postconsultation, in the same manner as in the TAU phase. This exit survey had the same questions as those in the TAU phase, with additional items related to Check Up GP as described in Table 2: acceptability, usability, overall opinion, and willingness to use again in the future.

#### Phase 3: Postintervention

At the end of the exit survey, participants were invited to participate in a follow-up postintervention interview held within 2 weeks of their consultation at a mutually agreed time. The interview guide was informed in part by the themes emerging from the codesign workshops [41]. The aim of these interviews was to explore young people’s experience of using Check Up GP, including how it affected the consultation and their relationship with their GP, and whether they would like to use the app again in the future.

### Reimbursement

Each patient who completed an exit survey received an Aus $10 gift voucher. Youth participants who were interviewed received an additional Aus $30 gift voucher. The clinic received Aus $2000 for their involvement. In addition, each receptionist received Aus $100 at the end of the study for the additional work required of them to administer the app.

### Analysis

Quantitative data from youth exit surveys were analyzed using SPSS version 23. Youth participant characteristics in the TAU and intervention groups were compared using chi-square test for independence. Where assumptions of expected cell frequency were not met, Fisher exact test was used. Data from all scaled variables showed major deviation from the normal distribution. Transformation did not adequately improve normality, so the nonparametric Mann-Whitney *U* analysis was used to compare groups for all variables. The Mann-Whitney *U* test has the added advantage of being suitable for uneven sample sizes. In comparing disclosure ratings between groups, “not applicable” responses were not included in the analysis. Scores for patient-centered care items were combined into five core components of the patient-centered model as described by Little et al [16]: communication and partnership, personal relationship, health promotion, positive and clear approach to problem, and interest in effect on life. Items for disclosure and patient-centered care were also combined to create a total disclosure and patient-centered care score. Internal consistencies of the following were calculated using Cronbach alpha: items in each of the four patient-centered core factors, total patient-centered care, and total disclosure. Effect sizes for all Mann-Whitney *U* analyses were calculated and reported as *r* with magnitudes using Cohen criteria (.1=small, .3=medium, .5=large) [52].

**Figure 1:**
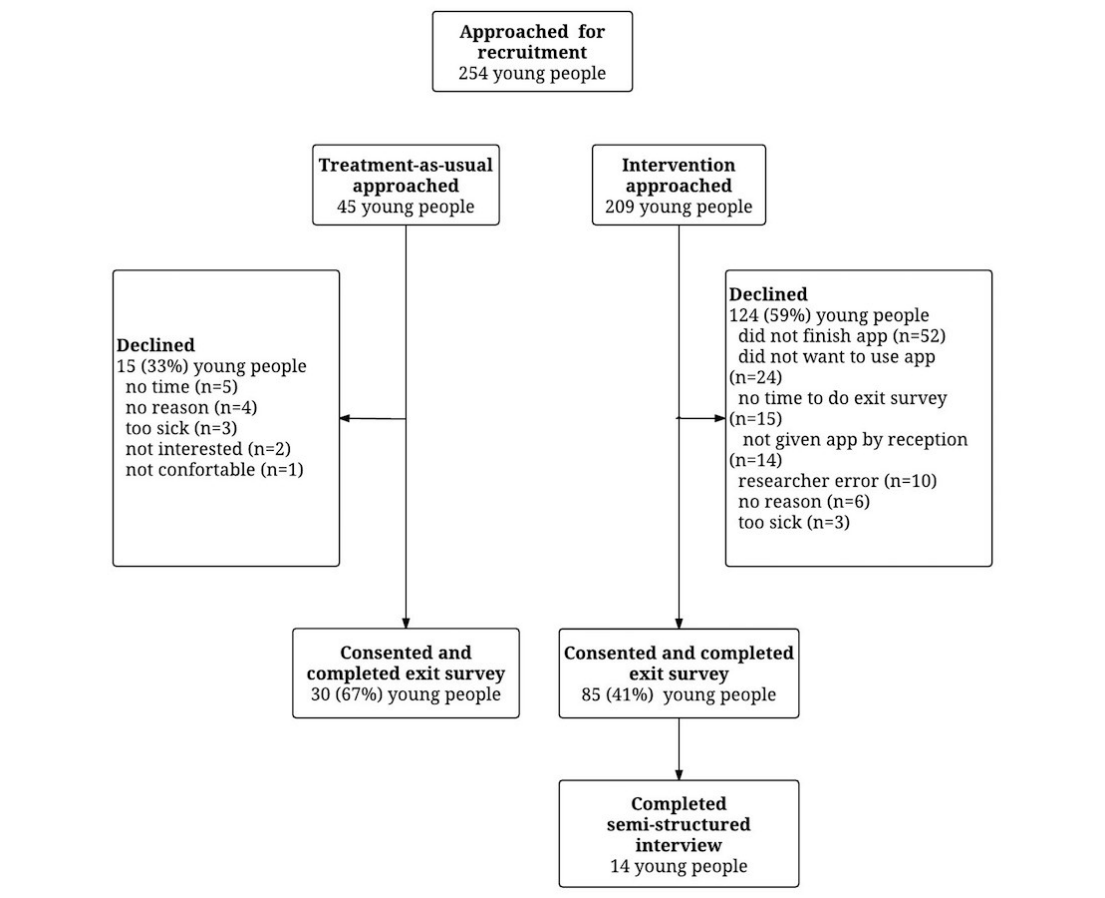
Recruitment breakdown of young people approached in the treatment-as-usual and intervention phases of the study.

Interviews were audio-recorded and transcribed verbatim by a professional transcription service. The first author compared the transcriptions to the audio recordings to ensure accuracy before coding all transcripts using NVivo 11 software. The second and third authors used this coding structure to independently code two transcripts. All authors then met to compare codes, revising the coding structure as required. The first author recoded all transcripts using the updated coding framework before conducting a thematic analysis [53]. These themes were discussed with all authors resulting in final themes.

## Results

### Participant Characteristics: Quantitative Phase

All four GPs were male and reported having had previous training in adolescent health. As shown in Table 3, each GP rated their enthusiasm for seeing a young person highly, with a mean rating of 9.5 (SD 1.3) out of 11. The participating GPs also highly rated their knowledge and confidence in consulting and communicating with young people, and exploring issues beyond the presenting problem.

Figure 1 shows the breakdown of young people who were approached and participated in the TAU and intervention phases of the study. In the TAU phase, 45 young patients were approached, of whom 30 (67%) agreed to participate. In the intervention phase, 209 patients were invited to participate, of whom 85 (40.7%) agreed. Of the 124 patients (59.3%) who declined to participate, 15 had completed Check Up GP, but did not have time to complete the exit survey.

**Table 3:** General practitioner (GP) self-rated enthusiasm, and knowledge and confidence of consulting and communicating with young people.

GP characteristics		GP 1	GP 2	GP 3	GP 4	Mean (SD)
Age group (year range)		45-54	45-54	45-54	55-64	
Enthusiasm for seeing young people (max=11)		10	8	9	11	9.5 (1.3)
**Consulting with young people (age 14-17) (max=7)**						
	Knowledgeable	5	5	6	7	5.8 (1.0)
	Confident	6	4	5	7	5.5 (1.3)
**Consulting with young people (age 18-25) (max=7)**						
	Knowledgeable	5	5	6	7	5.8 (1.0)
	Confident	6	5	6	7	6.0 (.8)
**Consulting with male young people (max=7)**						
	Knowledgeable	5	5	6	7	5.8 (1.0)
	Confident	6	5	5	7	5.8 (1.0)
**Consulting with female young people (max=7)**						
	Knowledgeable	5	5	5	7	5.5 (1.0)
	Confident	6	4	5	7	5.5 (1.3)
**Communicating with young people (max=7)**						
	Knowledgeable	5	5	5	7	5.5 (1.0)
	Confident	6	4	5	7	5.5 (1.3)
**Exploring issues beyond presenting problem (max=7)**						
	Knowledgeable	6	4	6	7	5.8 (1.3)
	Confident	6	3	4	7	5.0 (1.8)

**Table 4:** Characteristics of youth participants in the treatment-as-usual (TAU) (n=30) and intervention (n=85) phase.

Youth characteristics		TAU (n=30)	Intervention (n=85)	Group comparison^a^	
				χ^2^_1_	*P*
**Gender, n (%)**				0.8	.38
	Male	11 (37)	39 (46)		
	Female	19 (63)	46 (54)		
Age (years), mean (SD)		19.13 (2.62)	19.93 (3.32)		
**Age range (years)**		14-23	14-25	0.2	.66
	14-17	9 (30)	22 (26)		
	18-25	21 (70)	63 (74)		
**Sexuality, n (%)**					
	Heterosexual	27 (90)	83 (97)		.20
	Bisexual		1 (1)		
	Questioning	2 (7)	2 (2)		
	Other	1 (3)			
**Activities, n (%)**					.19
	Full-time work	3 (10)	16 (19)		
	Part-time work		10 (12)		
	Unemployed		2 (2)		
	Home duties		1 (1)		
	Have job, but not there due to illness		1 (1)		
	Student attending school	9 (30)	22 (26)		
	Student attending university	18 (60)	33 (33)		
**First time at clinic, n (%)**					.28
	Yes	2 (7)	2 (2)		
	No	28 (93)	83 (98)		
**First time with doctor, n (%)**					.56
	Yes	6 (20)	12 (14)		
	No	24 (80)	73 (86)		
**Attending with parent, n (%)**					.20
	Yes, went into consult with young person	12 (40)	27 (31)		
	Yes, but remained in waiting room		8 (9)		
	No	18 (60)	51 (59)		

^a^Those comparisons that were not chi-square values were calculated with Fisher exact test.

Demographic characteristics of youth participants who completed the quantitative exit survey are presented in Table 4. There were no differences between the characteristics of patients in the TAU group and those in the intervention group.

### Participant Characteristics: Qualitative Interviews

Fourteen youth participants from the intervention phase participated in qualitative interviews. Of those interviewed, nine were female and five were male; three participants were aged 14 to 17 years and 11 were aged 18 to 25 years (mean 20.9, SD 3.4 years). Eight participants were university or college students, two were at high school, three in full-time employment, and one was unemployed. Five of the 14 attended the clinic with a parent, with two of these five young people reporting their parent went into the consultation with them.

### Quantitative Findings

Young people’s median ratings of disclosure of health and lifestyle issues are shown in Table 5, with higher ratings indicating greater disclosure. Compared to the TAU group, young people using Check Up GP reported higher ratings of disclosure on all health and lifestyle issues, except for physical health. Median ratings of disclosure for four issues (alcohol/other drugs, sexual health, sexuality, and hurt self/others) improved from the minimum rating (1/5) in TAU to the maximum rating (5/5) in the intervention group. Medium effect sizes were found for all domains, except for sexual activity, sexuality, and diet that each had small effect sizes.

The items for each of the five core components of patient-centered care had high internal consistency. The Cronbach alpha coefficients were .99 for communication and partnership, .97 for personal relationship, .96 for health promotion, .97 for positive and clear approach to problem, and .89 for interest in effect on life.

Young people’s median ratings of patient-centered care are shown in Table 6, with lower ratings indicating greater patient-centered care. Compared to those in the TAU group, young people in the intervention group rated patient-centered care better (lower median ratings) in four of the five core factors of patient-centered care: communication and partnership, personal relationship, health promotion, and interest in effect on life. Effect sizes were small for each, except for “interest in effect on life,” which had a medium effect size. There was no difference between TAU and intervention groups for positive and clear approach to the problem.

There were no overall differences between TAU and intervention groups for patients’ ratings of youth-friendly care: how comfortable they felt, the level of trust in the GP, the extent to which their service met their expectations, and whether they had enough time to ask the GP everything they wanted to. Young people in the intervention group had lower ratings for “I would lose control of my emotions” (z=–2.087, *P*=.04) compared to those in the TAU group. The effect size was small (*r*=.19). There were no other differences in rating of judgment.

**Table 5:** Youth disclosure ratings of health and lifestyle issues in the treatment-as-usual (TAU) and intervention phase.

Health domain	TAU		Intervention		*U*		z		*P*		*r*	
	Median (IQR)	n	Median (IQR)	n								
Physical health	5.0 (2.0)	30	5.0 (2.0)	80		1042.50		–1.259		.21		.12
Home life	2.0 (1.0)	25	4.0 (3.0)	76		487.50		–3.789		<.001		.38
School/work	2.0 (2.0)	27	4.0 (2.0)	81		523.50		–4.188		<.001		.41
Bullying	1.0 (0)	22	3.5 (4.0)	56		312.50		–3.663		<.001		.41
Alcohol/other drugs	1.0 (0)	24	5.0 (3.0)	68		411.00		–3.839		<.001		.40
Sexual health	1.0 (2.0)	25	5.0 (4.0)	69		627.00		–2.168		.03		.22
Sexuality	1.0 (1.0)	24	5.0 (4.0)	66		548.00		–2.463		.01		.23
Hurt self/others	1.0 (0)	21	5.0 (4.0)	63		337.00		–3.717		<.001		.41
Mood	2.0 (1.0)	27	4.0 (2.0)	78		517.50		–4.142		<.001		.40
Addiction	1.0 (0)	19	4.5 (4.0)	58		256.50		–3.756		<.001		.43
Stressful events	2.0 (2.0)	28	4.0 (3.0)	80		611.50		–3.732		<.001		.36
Diet	3.0 (2.0)	27	4.0 (4.0)	75		706.50		–2.417		.02		.24
Exercise	2.5 (3.0)	26	5.0 (4.0)	75		607.50		–3.044		.002		.30
Sleep	3.0 (2.0)	27	4.0 (2.0)	81		681.50		–3.069		.002		.31
Risky behavior	1.0 (0)	23	2.5 (4.0)	66		475.00		–2.923		.003		.31
Safety	1.0 (0)	22	3.0 (4.0)	65		422.00		–3.129		.002		.34
Total^a^	27.5 (11.0)	30	44.5 (45.0)	84		648.50		–3.938		<.001		.37

^a^Cronbach alpha=.968.

**Table 6:** Reported ratings of patient-centered care by patients in treatment-as-usual (TAU) (n=30) and intervention groups (n=85).

Patient-centered care components	TAU, median (IQR)	Intervention, median (IQR)	*U*	z	*P*	*r*
Communication and partnership	26.5 (44.5)	12.0 (14.0)	849.00	–2.794	.01	.26
Personal relationship	9.5 (9.5)	4.0 (7.5)	880.50	–2.605	.01	.24
Health promotion	7.5 (5.8)	4.0 (6.0)	944.50	–2.175	.03	.20
Positive and clear approach to problem	10.5 (12.3)	5.0 (9.0)	991.00	–1.851	.06	.23
Interest in effect on life	7.0 (5.3)	3.0 (4.5)	734.50	–3.550	<.001	.33
Total^a^	54.5 (69.0)	29.0 (35)	801.50	–3.030	.002	.28

^a^Cronbach alpha=.991.

**Table 7:** Youth experience and acceptability of using Check Up GP.

Youth experience and acceptability	Not at all, n (%)	Only a little bit, n (%)	Somewhat, n (%)	Quite a bit, n (%)	Very much, n (%)
How confident are you that Check Up GP was able to provide an accurate picture of yourself to your GP?	2 (2)	4 (5)	31 (37)	37 (44)	11 (13)
How comfortable were you disclosing personal information through Check Up GP?	0	10 (12)	23 (27)	24 (28)	28 (33)
Were the questions in Check Up GP difficult to understand?	55 (65)	15 (18)	11 (13)	4 (5)	0
Did any of the questions in Check Up GP cause you to become upset?	66 (78)	10 (12)	6 (7)	1 (1)	2 (2)
To what extent did you feel your GP address the issues raised in CUGP?	3 (4)	7 (8)	17 (28)	32 (38)	26 (31)

Check Up GP was highly acceptable to patients. In all, 86% (73/85) thought the app was a “good idea,” 13% (11/85) felt unsure (“don’t know”), and only 1% (1/85) thought it was a “bad idea.” Most young people (63/85, 74%) wanted to use Check Up GP again, and only 6% (5/85) did not. Of those who wanted to use Check Up GP again, and responded to the question, more than half (36/62, 58%) wanted to use it twice a year or more, 15% (9/62) wanted to use it once a year, and a further 23% (14/62) wanted to use the app every time they saw their GP.

Table 7 shows the experience and acceptance by patients using Check Up GP. The majority of patients (48/85, 56%) were “very much” or “quite a bit” confident that the app provided an accurate picture of them to their GP. The majority of patients (52/85, 61%) were also either “very much” or “quite a bit” comfortable about disclosing personal information about themselves through Check Up GP. Most patients (58/85, 68%) reported that their GP addressed issues raised by Check Up GP “quite a bit” or “very much.” Almost all (76/85, 89%) reported that the questions caused them to become upset either “not at all” or “only a little bit.” In all, 82% of patients (70/85) rated the questions as being difficult to understand either “not at all” or “only a little bit.”

The usability and design of Check Up GP was also highly rated by patients. In all, 95% (81/85) rated Check Up GP as either being “able to use immediately” or “easy to learn”’ and 75% (64/85) rated Check Up GP as having either a “high level of visual appeal” or being “very attractive, memorable.”

### Qualitative Findings

Our analysis of interviews with participants found four main themes: identifying unmet needs, creating scope, moderating factors for use, and future use.

#### Identifying Unmet Health Needs

Many participants reported that using Check Up GP helped them to identify and disclose issues that they had not previously discussed, or thought would be relevant to discuss, with their GP:

It definitely brought things up that wouldn’t normally have been discussed in a consult...I feel like even the fact that those questions are being asked about your sleeping habits, your alcohol consumption, your drug use; the fact that they’re being asked in a health context at all could be beneficial.

Male, 24

In particular, participants reported that using Check Up GP made it easier to disclose sensitive issues, even though they knew the responses would be available to their GP. These young people “wouldn’t really like to tell the doctor in person...I’ve just always felt more comfortable with [technology]” (female, 19 years). There was a recognition that once the difficulty of disclosure could be overcome, problems could then be addressed:

I’ve wanted to bring up [mental health issues] with my doctor before and haven’t because I hadn’t really had the platform and felt a bit nervous about it.

Male, 23

In addition to facilitating disclosure, it was felt that using Check Up GP enabled participants to go into the consultation feeling more prepared and in control, and knowing what topics were going to be discussed. Check Up GP allowed them to “indicate to your doctor that this is what I’m going to talk to you about and this is where I’m heading” (female, 18 years). Thus, a shared understanding of what would be focused on in the consultation was achieved through the app in a timely manner:

You and the GP are obviously on a similar understanding, and then you can get right into whatever your concerns are.

Female, 17

#### Creating Scope for Addressing Unmet Health Needs

As well as facilitating the disclosure of unmet health needs, using Check Up GP created scope for addressing these needs within the consultation:

[The GP] took my responses...and gave a good reason to keep that [behavior] in check. Which I thought was good. It wasn’t just tick, tick, tick, it’s like he used it to explore further about my lifestyle choices and he did ask probing questions.

Male, 24

Although participants were generally very satisfied with how their GP addressed their concerns, a few felt they did not have sufficient time because their GP was either running well behind schedule or their responses were only addressed at the very end of their consultation. However, these participants also reported that their GP invited issues to be discussed at a future time:

We didn’t really address any of the problems in detail because it was towards the end of the consultation and he was going over time. But he said at the end, “If you do ever want to talk about any of these, you’re more than welcome to.”

Female, 23

A positive consequence of discussing unmet needs was that many participants felt that their GP had improved their understanding of the young person’s life.

When you go in sometimes you just focus on one question. So I think it was good to have a bit more of a general understanding.

Female, 24

Indeed, the process of unpacking responses led to some participants feeling a greater sense of connection and rapport with the GP. These young people felt that their GP showed a genuine interest and “actually care about what’s happening” (female, 24 years) in their lives:

They’re not just saying “how’s it going,” they’re sitting there and asking you about things that might be concerning you.

Male, 23

In addition to addressing unmet needs, the two participants who went into the consultation with a parent reported that the GP asked for time alone. One participant reported that this was the first time that time without parents had been requested: “I’ve never had that before, so that was good” (female, 15 years).

Only one participant reported having a negative experience of using Check Up GP. This young person felt their GP did not adequately attempt to explore the issues raised due to a lack of time:

He just said “I don’t think I need to lecture you on drugs and alcohol”...I just thought well what’s the point of me putting it in there if I’m going to get a bit of a judgy comment about it rather than in an endeavor to understand a bit more to see if it’s worth talking about...if he’d had more time then he would have gone into that a bit more.

Male, 23

#### Moderating Factors of Use

We discovered three factors that moderated use of the tool: young people’s motivation and honesty, the content and functionality of the tool, and administration.

All participants recognized the potential value of the tool, even those who had no current issues or had a good relationship with their doctor:

Perhaps it doesn’t necessarily apply to me, but I can see it working really well for other people, or at least it didn’t apply to me that time. Who knows in the future?

Male, 19

Four of the five males interviewed, along with one of the nine females, volunteered that Check Up GP gave them something useful to do in the waiting room:

I get bored very easily, especially in the waiting room, and if I have something to do that is actually relevant, it definitely will be a big help.

Male, 16

Almost all participants reported they answered questions honestly because they understood that disclosing was the aim of the app: “I wouldn’t lie because I think I’m only hurting myself if I’m lying to the GP because then he’s not going to be able to help me” (male, 24 years).

In contrast, five young people explained that there may be occasions when they would lie or fail to disclose the full extent of a behavior: if they were uncomfortable discussing an issue (such as sexting) or if they believed it did not affect their health:

Anything that I felt comfortable telling him, I definitely told the truth about. But there were maybe one or two things that I probably didn’t want to discuss with him that I just chose not to.

Female, 23

Another moderating factor to using Check Up GP was its content and functionality. Although participants felt there was a “good range of questions to get a general idea on someone” (female, 18 years), the language of the questions could be also perceived as “very direct and people might not want to answer that truthfully” (female, 24 years). Also, for a few participants, the length was “a little bit long because if the doctor was running on time, you probably wouldn’t have enough time to finish it” (female, 25 years).

We found that the context of using Check Up GP could be an important factor influencing young people’s engagement with the app. Although all young people felt they had sufficient privacy when completing it in the waiting room, a few participants also expressed the concern that their responses would be influenced if a parent or family member was sitting next to them:

If I’d been sitting next to a parent or family member, and they’d been looking over my shoulder—people curious [about] what it is—I think that would influence a lot of people’s answers, my own included.

Male, 19

Many participants felt they had received insufficient explanation about Check Up GP by receptionists. Instead, most relied on the information provided at the start of the tool or by the researcher to understand the app’s purpose and how the information would be used by GPs:

Reception didn’t really say much about it, which was a bit weird. But on the tool the front page gave it a pretty decent, pretty lengthy explanation (male, 19 years).

#### Future Use

All participants reported that they wanted to use Check Up GP regularly, when they see their GP one or two times per year. They felt that this would enable new issues that emerge to be identified and discussed:

[Twice a year] seems like the right space of time where things can change, life events happen, you know, relationships, or family members going through difficulties, yourself going through difficulties, employment changing.

Male, 19

Participants felt regular use of Check Up GP would also ensure that issues that had emerged previously could be revisited to “see how I’m tracking” (female, 23 years).

Finally, participants suggested a number of ways that the Check Up GP app could be developed. One suggestion was adding the ability to view previous answers. Another was the ability to receive automated, personalized health information, such as on healthy diet or exercise.

## Discussion

### Principal Results

This mixed methods study aimed to investigate whether the implementation of a health and lifestyle-screening app, Check Up GP, codesigned by young people and GP staff and grounded in the theories of patient-centered and youth-friendly care, improved disclosure of health risks and care experiences of young patients attending general practice. We were also interested in understanding young people’s acceptance of and experience with using a screening app as part of their routine health care with their GP.

Overall, we found using Check Up GP improved disclosure across all health and lifestyle domains, including sexual health, alcohol and other drugs, and risky behavior, except for physical health where disclosure was already high. This supports previous research showing that the use of technology improves young people’s disclosure of sensitive health issues [24,25]. Our qualitative interviews suggested that using Check Up GP not only led to identification of unmet needs, but created scope for discussing and addressing these needs in a way that does not usually occur for young people during routine care. It is encouraging that the majority of young people (75/85, 88%) felt the issues raised were at least somewhat addressed, particularly given previous research has found that GPs ignored certain domains identified as issues by young people [54]. It is possible that the functionality of Check Up GP for the GPs (eg, traffic-light summary report with suggested actions and referral options), in addition to the emphasis in GPs’ training on the need to follow up on all raised issues, facilitated GPs in our study to more consistently address raised issues. It is also possible that the GPs in this study felt particularly engaged and skilled to address issues, and willing to spend extra time doing this.

Young people who used Check Up GP felt more prepared to discuss the issues raised in their consultation. In our quantitative findings, young people using the app were less concerned that they would lose control of their emotions than those in the TAU group. This finding was reflected in our interviews with young people, with participants appreciating that the tool provided them a chance to prepare and reflect on their responses before the consultation. In doing so, young people felt more emotionally prepared to have a conversation about sensitive issues than they otherwise would have been. Although similar results were found with young people using a screening tool with a nurse in a school setting [55], this is the first study to our knowledge that explores young people’s experience of the tool in the general practice setting, outside of well-child visits.

Using Check Up GP had a profound impact on young people’s perception of patient-centered care. These findings support and extend previous research that had only measured and found improvements in one element of communication [34]. As well as communication and partnership, we found that using Check Up GP improved personal relationships, health promotion, and interest in the effect of the problem on life. In contrast, Gadomski et al [33] only found improvements in doctor engagement and no change in partnership or rapport. This difference may be due to measuring patient-centered care by analyzing audio recordings of consultations, rather than patient’s own ratings. Another recently published study found no changes in communication and experience of care, or in health information [56]. However, both of these studies were conducted with young people at well visits, which are not well-attended by key subgroups of young people such as low income and uninsured [14]. Thus, our findings suggest that the use of a technology-based screening tool has the potential to transform young people’s experience of care.

In contrast to patient-centered care, using Check Up GP did not improve core indicators of youth-friendly care. We found no difference between TAU and intervention groups for young people’s assessment of trust, whether they had enough time to ask the GP everything they wanted to, their level of comfort, nor the extent to which the service met their expectations. There was also no change on all but one measure of judgmental reactions. The lack of improvement may be due to a ceiling effect, with these variables being rated very highly in the TAU group. Indeed, a previous randomized controlled trial of a paper-based screening tool also found youth participants rated their level of trust in their GP highly in both control and intervention groups [57]. Perhaps practices volunteering for these studies are already interested in meeting the needs of young people and therefore have high baseline scores. That there was almost no negative effect on youth-friendly variables in our study is a positive finding and suggests that this technology can be integrated into routine care and can direct focus away from the presenting issue without necessarily undermining the delivery of quality youth-friendly care. However, further research is needed to determine whether factors other than the ceiling effect or participating GP characteristics are responsible for the nonsignificant differences in youth-friendly care.

There are a number of established theories that may explain the underlying causal mechanisms of our findings. The Culture of Situated Cognition theory proposes that cultural mindsets influence what feels fluent and what is engaged with [58]. Drawing on this theory, the patient decision aid theoretical framework developed by Alden and colleagues [59] proposes that patient screening and decision aids that are culturally targeted to specific cultural groups, using appropriate imagery, evidential information, linguistics, and values relevant to that group, will enhance feelings of congruency with the material, thereby increasing “processing fluency.” Improved processing fluency in turn predicts enhanced preparedness (eg, confidence and openness to participating) leading to improved outcomes. In addition, in the context of technology and using the Stimulus-Organism-Response theory, Jiang and colleagues [60] demonstrated that interactive features or cues on websites trigger a greater cognitive and emotional involvement of users, which in turn elicit desired behaviors. Thus, these theories would predict that an interactive technology-based screening tool that is designed specifically for a youth subculture is likely to enhance processing fluency and increase engagement, resulting in increased disclosure and perceptions of patient-centered care. Further research is needed to further explore the underlying causal mechanisms with this technology.

A particularly interesting finding from the qualitative interviews was the dynamic nature of honesty in participants’ responses on Check Up GP. For these young people, honesty was not a binary but was fluid, considered and controlled by the young person. Indeed, a substantial minority (31/85, 37%) reported that Check Up GP only “somewhat” provided an accurate picture of them. Given this was the first time participants had used such a tool, it may be that, understandably, young people wanted to “test the water” with their GP, to ensure any disclosure would be addressed with sensitivity. Alternatively, it may be that some participants were not yet ready to acknowledge they had a problem or to change their behavior. Perhaps using an app, such as Check Up GP, has the potential to be a catalyst for young people to reflect on their health and lifestyle and to begin the process of behavior change.

A positive finding of this study was the high proportion of young people who not only assessed Check Up GP as a “good idea,” but who also wanted to continue to use it at least once a year as part of their routine care. Although best practice guidelines recommend this type of screening to be done annually [20,21], and previous studies of technology-based [39] and paper-based [57] screening have also found a high acceptance rate, there has been no research on whether young people themselves want to be screened regularly for health and lifestyle issues. Our results suggest that young people see value in this kind of technology being integrated into their routine care with their GP.

Finally, our findings suggest that administration of the app may influence young people’s engagement with and use of Check Up GP. Most young people we interviewed felt that they did not receive adequate explanation from the clinic about Check Up GP. We also found that 42% (52/124) of young people who were not eligible to be included in the study were excluded because they did not have time to finish Check Up GP in the waiting room before their consultation. The introduction of technology into general practice needs to ensure all young people have the opportunity to use and benefit from it. There is now a solid body of research to suggest that the implementation of technology in health settings such as general practice is determined by interrelated individual, organizational, and social factors [61]. Further research is needed to understand how a screening app for young people can be successfully integrated within a general practice setting; another component of this study will address this theme in future work.

### Limitations

This study had a number of limitations. Because it was conducted at one clinic in a relatively socioeconomically advantaged area of one city, the generalizability of results may be limited. The four participating GPs were all male and of a similar age, all had previous adolescent health training, and all reported being highly confident and knowledgeable in consulting with young people. It is possible that GPs with less experience and enthusiasm for young people may not use and engage with Check Up GP in the same way as the GPs in this study, which in turn may influence young people’s experience of using it within their routine care. Young people in other socioeconomic areas may experience Check Up GP differently to those in this study. Another limitation of our study is that it did not have an experimental design and so did not have a randomized control group. Although we found no significant difference between the TAU and intervention groups in the quantitative data in key characteristics such as gender, age, and sexuality, it is possible that there were underlying and unidentified differences between the two groups.

### Future Research

This implementation study of a health and lifestyle-screening app for young people was conducted in one general practice clinic. Further research is needed to investigate the effect of using a health and lifestyle-screening app in a diverse range of clinic types and settings, and with a diverse range of GPs and youth participants. Future research could also investigate whether improvements found in this study are sustained over time and influence young people’s health and lifestyle. Finally, there is a need to explore the experience of PCPs and practice support staff in integrating Check Up GP into their clinical and administrative work, respectively; we will report on that phase of the study in the future.

### Conclusions

Previous research has found that using a technology-based health and lifestyle-screening tool as part of routine care can improve disclosure and communication with health professionals, which aids in early identification of issues and delivery of preventive health care. This study extends this research by providing new insights about the use and experience of using such an app by young people as part of their routine care in general practice. The results suggest that integrating a health and lifestyle-screening app into face-to-face regular care can enrich young people’s experience of seeing their GP, create scope to address unmet health needs, and become integrated into their regular health care.

## Appendix

Multimedia Appendix 1 App screen grabs of Check Up GP youth and general practitioner interface.

## Abbreviations

GP: general practitioner
PCP: primary care provider
SMS: short message service
TAU: treatment as usual
