# Supplementary material for: Improving Patient-Centered Care for Young People in General Practice With a Codesigned Screening App: Mixed Methods Study
Source: JMIR Mhealth Uhealth. 2017 Aug 11;5(8):e118. doi: 10.2196/mhealth.7816 (PMC5573432; doi:10.2196/mhealth.7816)
Supplement: Multimedia Appendix 1 [file mhealth_v5i8e118_app1.pdf]

Check Up GP: Youth interface

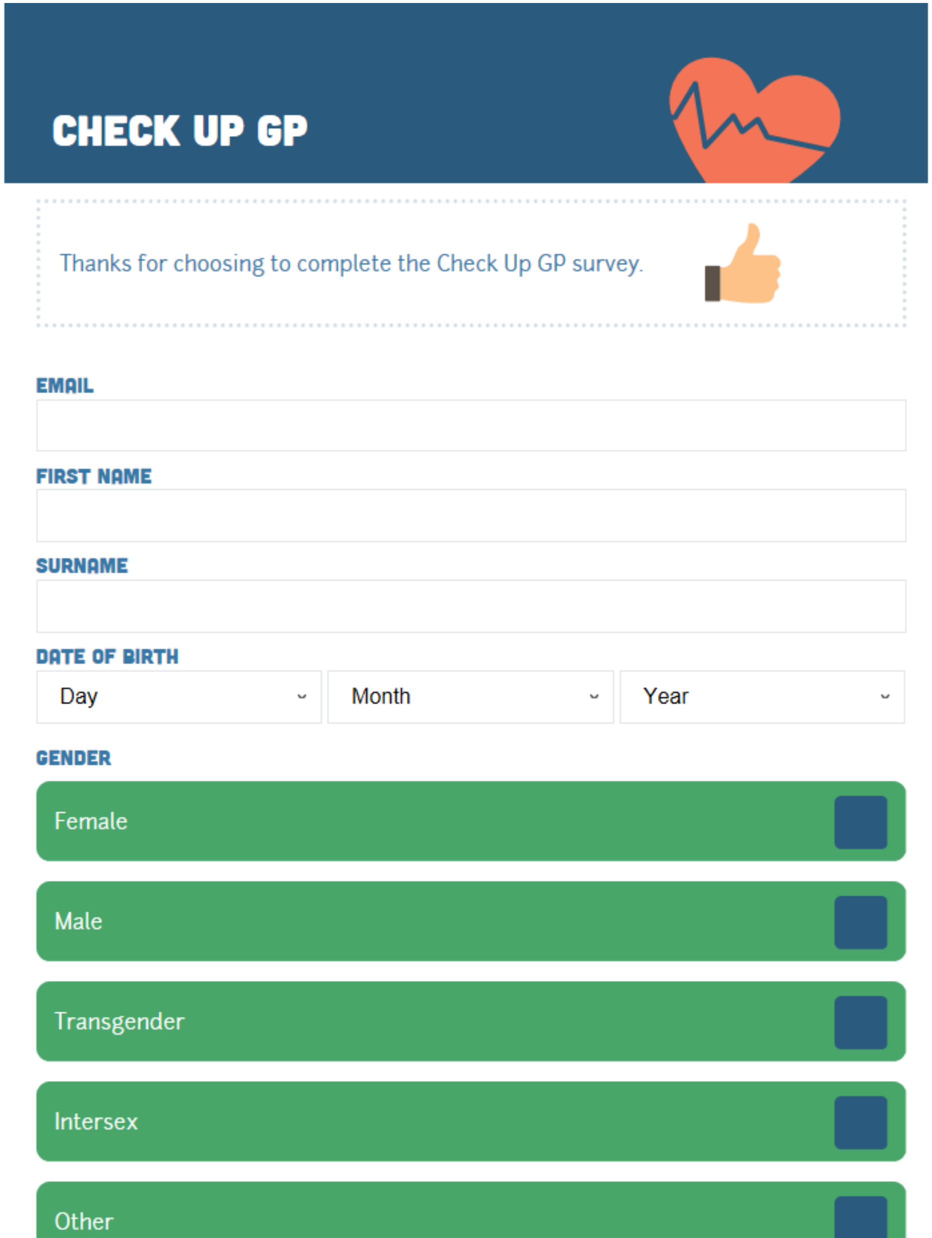

CHECK UP GP

Thanks for choosing to complete the Check Up GP survey.

EMAIL

FIRST NAME

SURNAME

DATE OF BIRTH

Day | Month | Year

GENDER

Female

Male

Transgender

Intersex

Other

Check Up GP: Youth interface

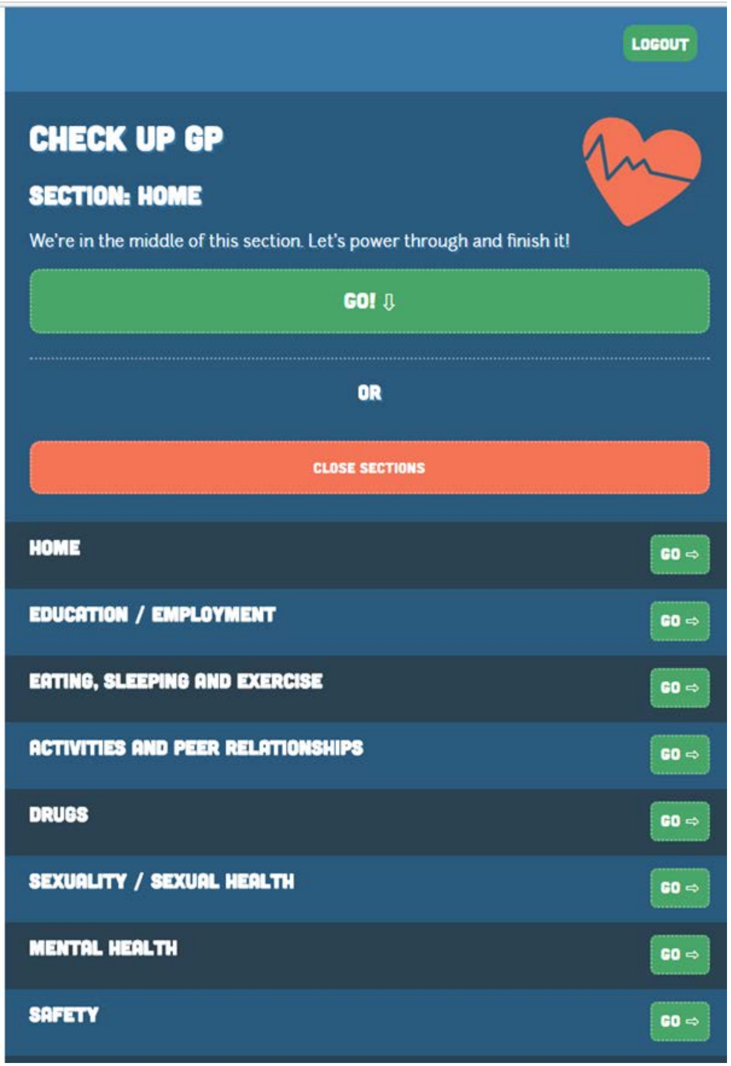

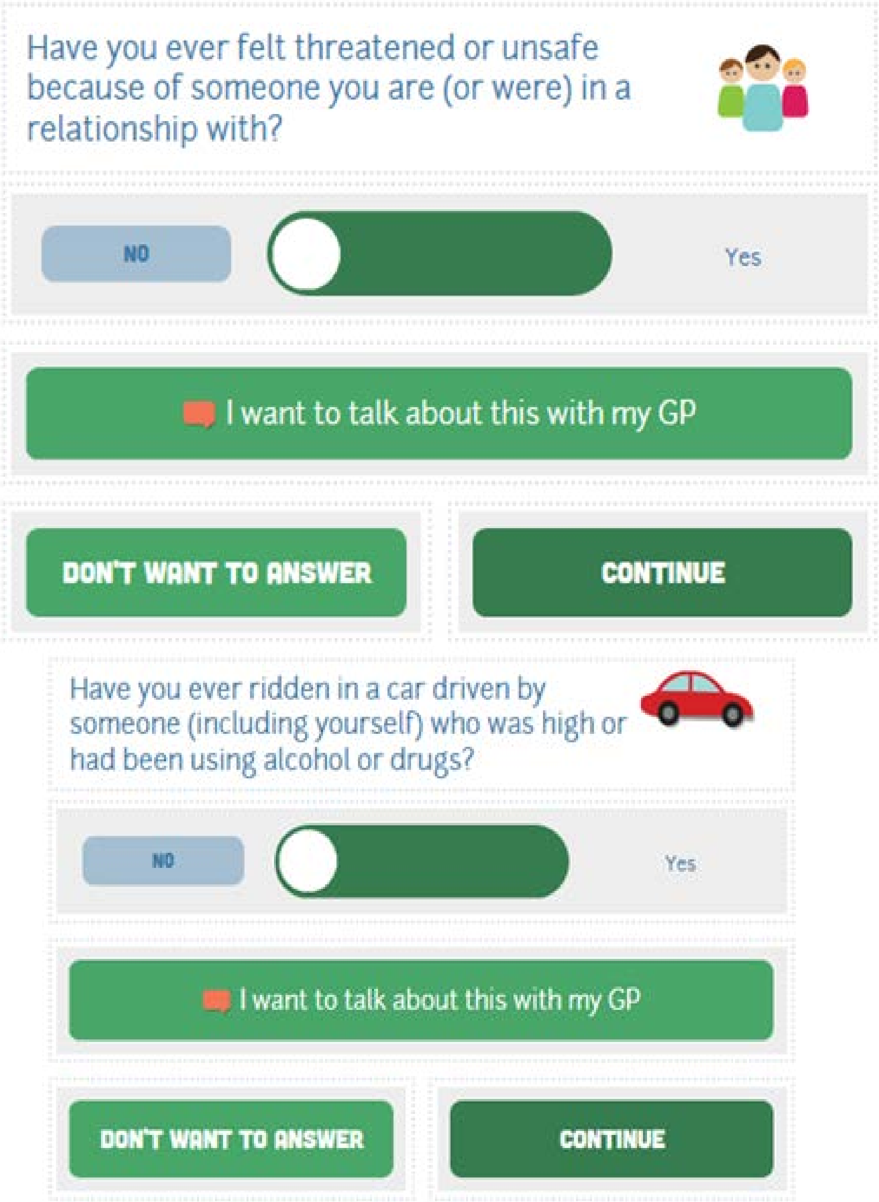

Check Up GP: GP interface

INDIVIDUAL REPORT PRINT | SAVE | IMPORTANT INFO | CLOSE

Name	Gender	D.O.B	Age	Sexuality	Results Gathered
Shaunagh O'Sullivan	Female	04/01/1992	24	Heterosexual / straight	6 Jun 2016

REPORT

CHECK FOR UPDATES

Expand All Collapse All

HOME	Concerning
EDUCATION / EMPLOYMENT	Very Concerning
EATING, SLEEPING and EXERCISE	OK
ACTIVITIES AND PEER RELATIONSHIPS	OK
DRUGS	OK
SEXUALITY / SEXUAL HEALTH	OK
MENTAL HEALTH	OK
SAFETY	OK
OTHER	OK

Check Up GP: GP interface

INDIVIDUAL REPORT PRINT | SAVE | IMPORTANT INFO | CLOSE

Name	Gender	D.O.B	Age	Sexuality	Results Gathered
Shaunagh O'Sullivan	Female	04/01/1992	24	Heterosexual / straight	6 Jun 2016

REPORT

CHECK FOR UPDATES

Expand All | Collapse All

HOME

SHOW: CONCERNING & FLAGGED ONLY   ALL ITEMS

Question	*Response*	*Suggested Action*
**4.** Have you experienced any of the following over the past 12 months (choose as many as applies to you):	• You started/finished uni or college<br>• A family member or close fried had (or has) a serious illness or injury	• Empathise and normalize<br>• Assess safety, explore coping strategies, sources of support<br>• Identify and reinforce strengths and protective factors (e.g. school connection, peer relations)<br>*Referrals*<br>• Info, tools to promote wellbeing and resilience: Biteback<br>• Lifeline 131 114, Kids Helpline 1800 55 1800, eheadspace.org.au 1800 650 890<br>• Counselling: headspace Camberwell 9006 6500<br>• Information and counselling: National Sexual Assault, Domestic and Family Violence Counselling Line 1800 737 732<br>• Guided meditation app: Smiling mind

EDUCATION / EMPLOYMENT

Very Concerning

EATING, SLEEPING and EXERCISE

OK
